# A Novel Approach for Temperature-Induced Ball Grid Array Collapse Observation

**DOI:** 10.3390/ma17112693

**Published:** 2024-06-02

**Authors:** Kristina Sorokina, Karel Dušek, David Bušek

**Affiliations:** Faculty of Electrical Engineering, Czech Technical University in Prague, 16627 Prague, Czech Republic; dusekk1@fel.cvut.cz (K.D.); busekd1@fel.cvut.cz (D.B.)

**Keywords:** electrical engineering, ball grid array, lead-free, thermomechanical analyzer, collapse, conductive bridge, microstructure

## Abstract

This study presents a new approach to investigating the impact of repeated reflow on the failure of ball grid array (BGA) packages. The issue with the BGA package collapse is that the repeated reflow can lead to short circuits, particularly for BGAs with a very fine pitch between leads. A novel approach was developed to measure the collapse of BGA solder balls during the melting and solidification process, enabling in situ measurements. The study focused on two types of solders: Sn63Pb37 as a reference, and the commonly used SAC305, with measurements taken at various temperatures. The BGA samples were subjected to three different heating/cooling cycles in a thermomechanical analyzer (TMA) at temperatures of 250 °C, 280 °C, and 300 °C, with a subsequent cooling down to 100 °C. The results obtained from the TMA indicated differences in the collapse behavior of both BGA solder alloys at various temperatures. Short circuits between neighboring leads (later confirmed by an X-ray analysis) were also recognizable on the TMA. The novel approach was successfully developed and applied, yielding clear insights into the behavior of solder balls during repeated reflow.

## 1. Introduction

In contemporary microelectronics, the trend of rapid miniaturization is prevalent, offering significant convenience in the everyday use of devices. Electronic components equipped with ball grid array (BGA) packages hold substantial potential in electronics assembly. However, the miniaturization of BGA components poses several challenges. With the reduced pitch between leads and repeated soldering, reliability issues can arise, such as the formation of conductive bridges (shorts) leading to non-functional devices. Moreover, a decreased pitch between leads can cause the faster growth of dendrites, which can ultimately result in device failure as was shown in studies [1,2,3,4,5,6].

Understanding the solder ball collapse during reflow soldering is crucial to achieving an adequate reliability of soldered BGAs. Inappropriate reflow conditions for components with small lead pitches can lead to the formation of conductive bridges, causing short circuits. For instance, a study [7] illustrated the challenges in finding an optimal temperature profile for soldering large BGA packages. Research has been conducted to study the formation of BGA solder joints during reflow. For example, one study [8] examined the shape of BGA solder balls after reflow at different temperatures and evaluated the influence of their shape on reliability. Other studies examined the collapse of the BGA solder joints after reflow. The collapse of BGA solder balls was analyzed by measuring the length of a diagonal line in a solder ball (edge–center–edge) [7] or by measuring the stand-off height after reflow [9]. Several works have also predicted the shape (geometry) of a BGA ball after one or several reflows using the finite element method [10,11,12,13,14,15] or computational fluid dynamic simulation [16]. There have also been studies examining mixed assemblies; for example, an assembly consisting of an SAC305 BGA solder ball with a printed Sn-Bi solder alloy [17] or a Cu BGA ball covered with an SAC305 solder alloy [18]. The initial collapse was evaluated after soldering; however, it is worth noting that the level of collapse with mixed assemblies, which was found to be low [17,18], may occur due to causes other than those studied in this work.

Although the previous research into BGA solder ball collapses has explored similar topics to this work, there has been no investigation into the in situ recording of BGA solder ball collapse during reflow. The aim of this study is to present a new approaching to measure the in situ behavior of BGA solder balls using a specially designed sample. The approach also enables the measurement of CTE changes throughout subsequent thermal cycles.

## 2. Materials and Methods

**Figure 1:**
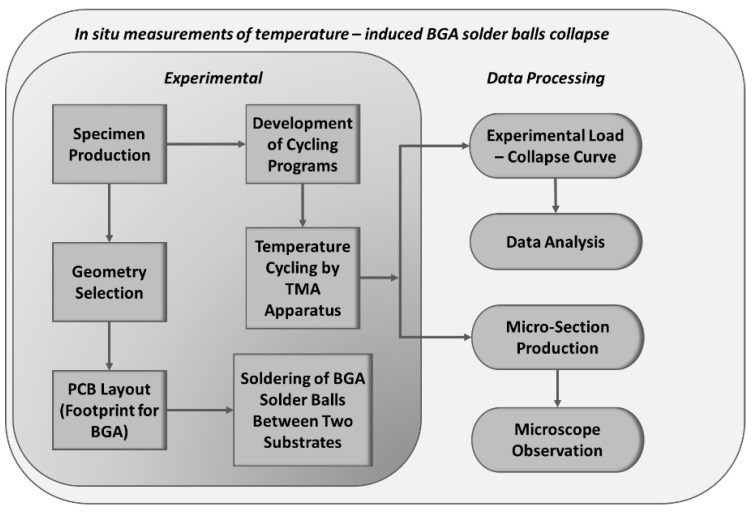
Detailed flowchart of the BGA solder balls’ collapse measurement process.

Detailed flowchart of the BGA solder ball measurement is shown in Figure 1. This flowchart outlines the step-by-step procedure used to measure the collapse of BGA solder balls. Each stage is clearly labeled to show the progression from sample preparation to the final analysis.

### 2.1. Sample Preparation

**Figure 2:**
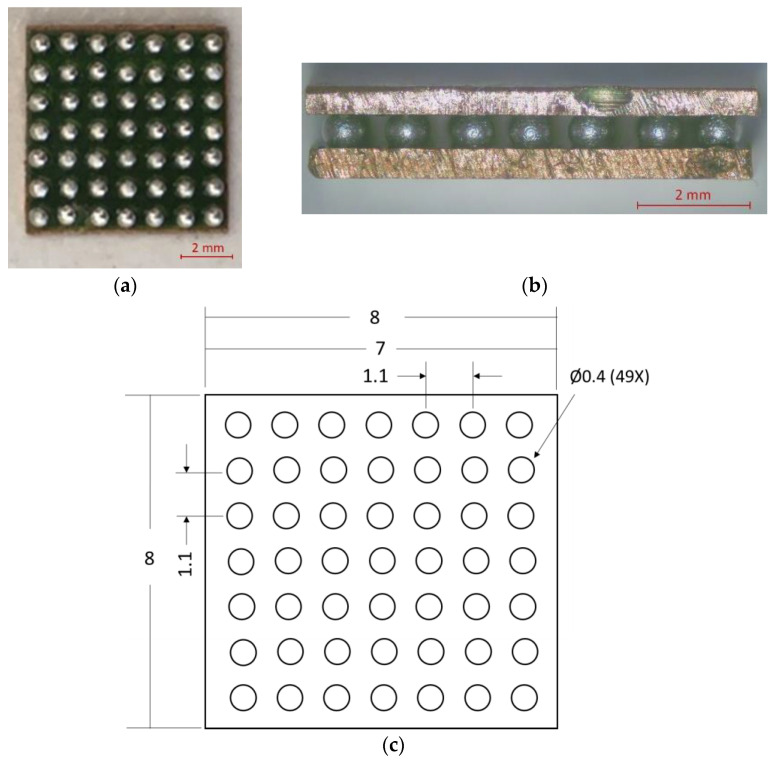
Stages of preparing BGA-like samples for TMA measurements: (**a**) initial placement of solder balls on one copper plate, (**b**) completion of BGA connections simulating the actual BGA setup, and (**c**) detailed view of solder mask grid of 49 contact pads with dimensions in mm.

BGA-like samples for TMA measurement (TA Instruments, New Castle, DE, USA) were made of two copper substrates and balls of solder alloy between them (see Figure 2a,b). Two copper plates were chosen as substrates primarily to eliminate the influence of the thermal expansion of the package or printed circuit board (PCB) and to avoid other factors, such as uneven weight distribution in the plastic/ceramic packages. The upper part, representing the BGA package, and the bottom part, representing the PCB, were constructed using identical copper substrates measuring 0.5 mm in thickness and 8 mm × 8 mm in size. This particular thickness was chosen to match the weight ratio of heavier BGA (metal package) components, where the pin-to-pin shorts are more common.

The copper plates were coated with a solder mask, creating a grid of 49 contact pads for solder balls (see Figure 2c). Each contact pad had a diameter of 0.4 mm and a pitch of 1.1 mm.

For the samples, solder balls with a diameter of 0.76 mm were chosen from two manufacturers for BGA reballing: Sn63Pb37 solder balls (Profound Material Technology Co., Ltd., Kaohsiung, Taiwan) and SAC305 solder balls (Shenzhen Xinhong New Electronic Tools Co., Ltd., Shenzhen, China). Solder balls were reflowed onto the copper pads of one copper substrate as on a BGA chip. This assembly was mounted on the second copper substrate and reflowed again. For both reflows, a no-clean paste flux PF 600 (Qualitek company, Addison, IL, USA) was applied to the copper pads beforehand. The MISTRAL 260 tunnel oven (Technoprint Ermelo, Ermelo, The Netherlands) with re-circulated hot air and three temperature zones were used for reflow. Two temperature profiles of trapezoidal type were used depending on the type of solder. Both solder profiles were chosen in the mid-range of the manufacturer’s recommendations and according to the IPC-J-STD-001 standard [19].

### 2.2. Measuring Approach

The collapse of the BGA solder balls was measured using a TMA apparatus (TA Instruments, New Castle, DE, USA) under a nitrogen atmosphere. Measuring the collapse of the solder balls in a liquid state under load and different temperatures provides information about the solder balls’ behavior during the reflow soldering process.

**Figure 3:**
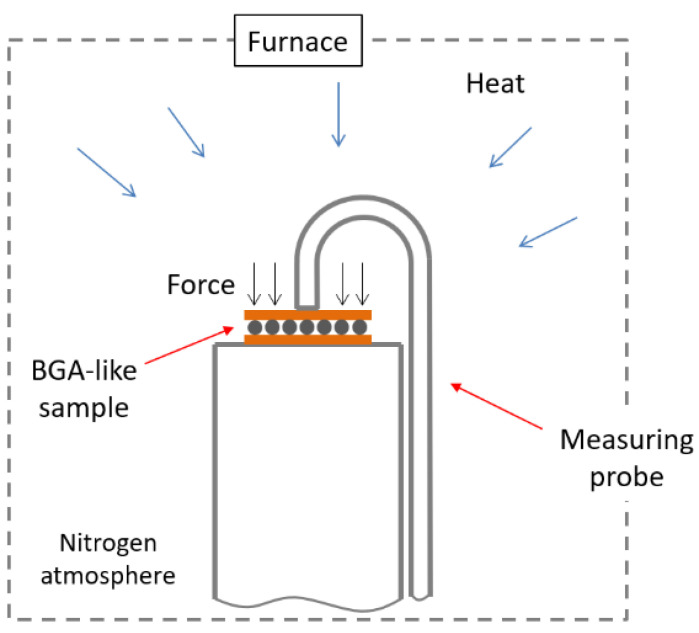
Schematic of the TMA setup for measuring BGA solder ball collapse using TMA apparatus.

The diagram in Figure 3 illustrates the configuration of the TMA apparatus, including the placement of the BGA-like package and the application of force. Three temperature programs were created. The programs consisted of three temperature cycles between 250 °C/280 °C/300 °C and 100 °C, accompanied by an additional force of 0.01 N that was applied to the sample in a perpendicular direction (see Figure 3). Such temperatures were chosen so that the lowest peak temperature is at least 20 °C above the melting point of any chosen solder alloy.

The temperature range between 250 °C and 300 °C was selected considering the commonly used values for SAC solders. The minimum peak temperature to obtain a solder joint is 230 °C. The maximum peak temperature used depends on various parameters, including the size of the PCB, its material, dimensions, assembled electronic components, etc. There may be a significant heat capacity difference across the PCB and, thus, different peak temperatures. To compensate for it, a longer temperature above liquidus (TAL) should be chosen according to the work by Pan J. et al. [20]. To ensure simultaneous solidification of all BGA solder balls, a slow controlled cooling rate of 5 °C/min was employed. For the Sn63Pb37 solder alloy, an additional program involving three temperature cycles at 220 °C was also implemented.

Initial heating started from ambient temperature, and the heating and cooling rates were identical for all three programs (5 °C/min). The measuring probe registered the dimensional changes of the BGA-like package (that is, the solder balls) during heating and cooling, enabling the evaluation of the solder behavior during melting. Verification measurements were performed on bare copper substrates to ensure that changes in substrate dimensions could be safely neglected.

## 3. Results

**Figure 4:**
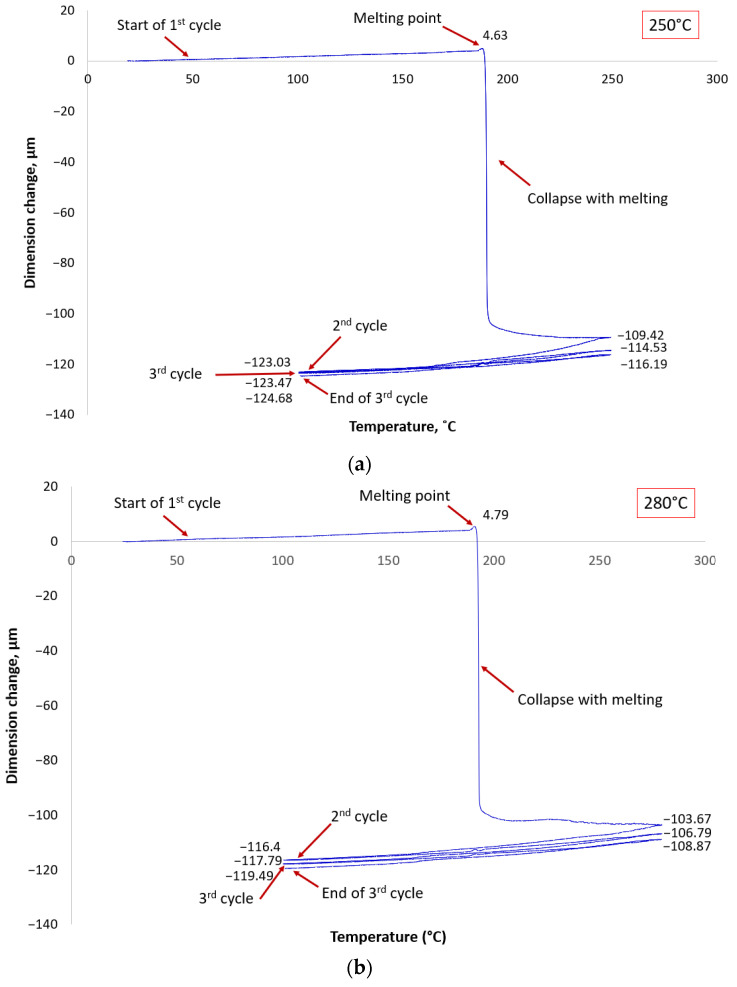

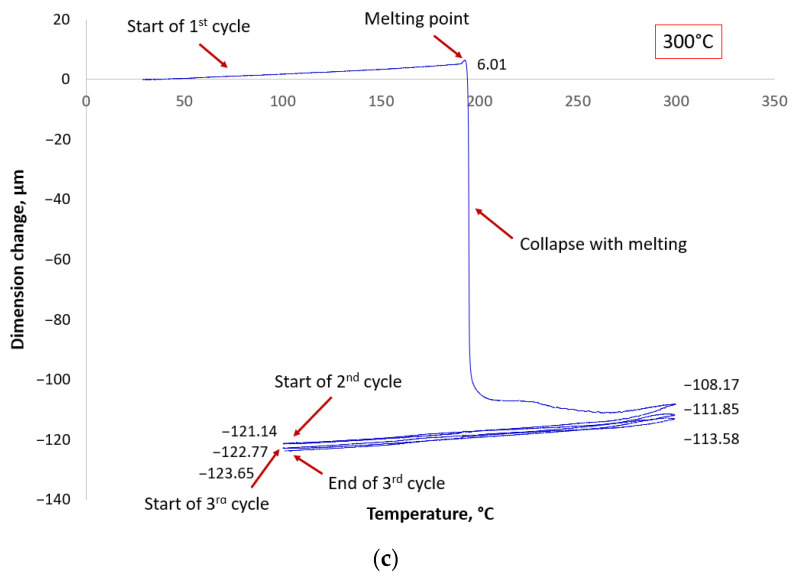
TMA measurement results showing the collapse behavior of SnPb solder balls at different temperatures. Each panel (**a**–**c**) demonstrates the solder ball collapse at 250 °C, 280 °C, and 300 °C, respectively.

The measuring probe recorded the movement of the upper plate during the cycles. The curves, plotted from the measured data, provided a clear representation of the behavior of the BGA solder balls during reflow. The collapse of the Sn63Pb37 BGA solder balls was consistent in all three temperature scenarios, despite significant temperature variations. In the first heating cycle, the molten Sn63Pb37 solder balls collapsed, indicated by a sharp drop in the graph line (see Figure 4a). However, during the second and third heating cycle, while crossing the melting point of the solder, no sharp drop in the line occurred, signifying no further collapse. This consistent behavior was observed at both higher temperatures, 280 °C and 300 °C (see Figure 4b,c).

**Figure 5:**
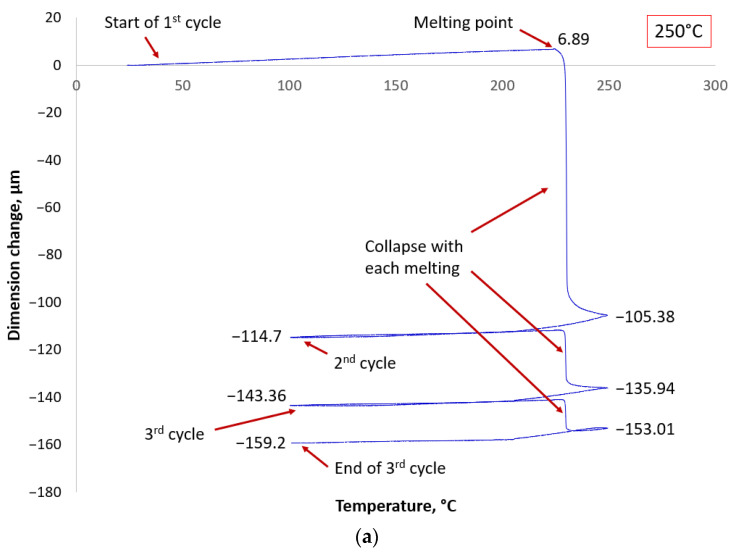

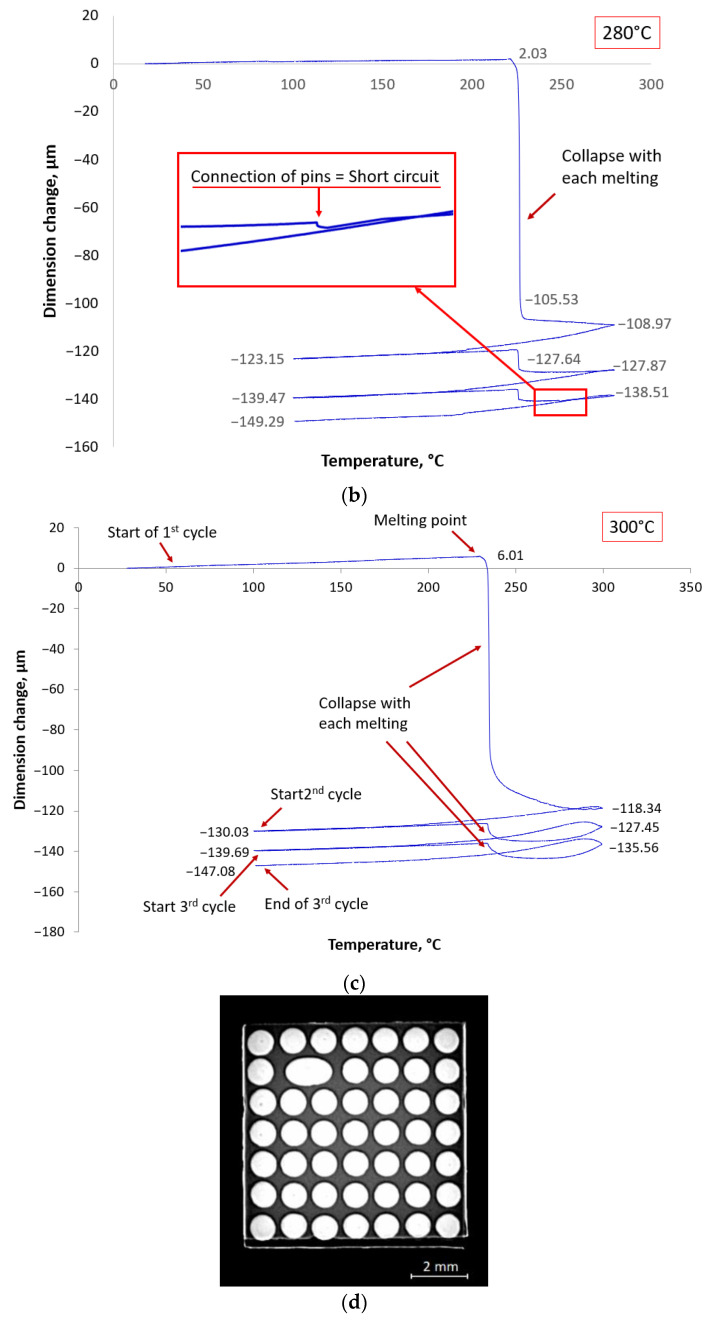
TMA measurement results showing the collapse behavior of SAC solder balls at temperatures (**a**) 250 °C, (**b**) 280 °C, and (**c**) 300 °C, respectively; and X-ray image (**d**) of BGA-like package with a short arisen at temperature 280 °C.

In the case of the SAC305 solder, a noticeable collapse of the solder balls occurred during each heating cycle. The TMA measurement at 250 °C is depicted in Figure 5a, where the solder balls collapsed after reaching the melting point in the first heating cycle, indicated by the initial drop in the line. Subsequently, during the second heating cycle, a noticeable but reduced collapse of the BGA solder balls took place. Additionally, during the third heating cycle, there is again a noticeable drop in the line, representing the further collapse of the BGA solder balls. This pattern is consistent with the measurements at 280 °C and 300 °C. The TMA measurements also revealed the interconnection of adjacent solder balls in a liquid state at 247 °C, as shown by the marginal drop in Figure 5b. An X-ray inspection confirmed the presence of shorted solder balls on the BGA sample (see Figure 5d).

It is worth noting that the magnitude of the collapse after the first cycle depends on the temperature program, with higher temperatures leading to a significantly larger initial collapse.

**Figure 6:**
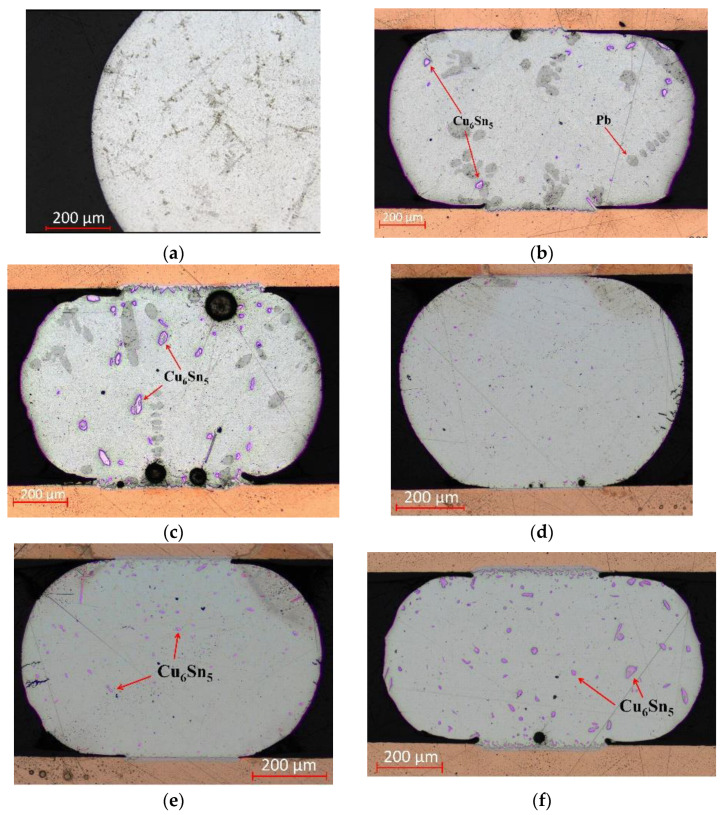
Microstructural changes in solder balls of (**a**) Sn63Pb37 with precipitated Cu_6_Sn_5_ grains after reflow, (**b**) after 1 cycle at 250 °C, and (**c**) after 1 cycle at 300 °C; and (**d**) SAC305 with precipitated Cu_6_Sn_5_ grains after reflow, (**e**) after 1 cycle at 250 °C, and (**f**) after 1 cycle at 300 °C.

The cross-section observation of the solder balls for both alloys (see Figure 6) revealed the changes in the BGA solder balls during the individual cycles. Higher temperatures and multiple heating cycles resulted in noticeable alterations to the solder’s microstructure. Elevated temperatures led to the growth of the Sn-Cu phase within the joint volume of both solder alloys. Additionally, the cross-sections showed that the grain number and its size increased with rising temperature (see Figure 6). The measured curves and cross-sectional images indicate that the growth of IMC could have contributed to the collapse behavior of the BGA solder balls.

## 4. Discussion

Using the novel approach described above, it becomes possible to predict the behavior of various solder ball alloys during reflow at different temperatures. For instance, the measured data showed that the collapse of the Sn63Pb37 BGA solder balls had a similar linear character for all three temperature scenarios (see Figure 4).

In contrast, the collapse of SAC305 BGA solder balls significantly increased with further cycles (see Figure 5).

As higher temperatures decrease the viscosity, as measured in the research [21], the initial collapse aligns with expectations—at higher temperatures, the collapse was more pronounced. The collapse of solder balls can also be explained by the dependence of surface tension on temperature. Referring to the research data in the literature [22,23,24], we can assume that the presence of a secondary and tertiary collapse in the SAC solder alloy while crossing the melting point (see Figure 5a,b), unlike the SnPb solder alloy, is due to its higher surface tension. Additionally, studies [22,23,24,25,26,27] have demonstrated that surface tension decreases with increasing temperature. This may explain the gradually decreasing secondary and tertiary collapses at temperatures of 250 °C, 280 °C, and 300 °C.

In addition to the lead-free SAC305 and leaded SnPb BGA-like samples, our experiment also investigated the collapse behavior of BGA-like samples featuring solder balls composed of lead-free Sn-Bi solder alloy. The solder mask grid used in these samples was identical to that of the aforementioned BGA-like samples. Notably, the thermo-mechanical analysis (TMA) results revealed that the collapse of Sn-Bi BGA solder balls during thermal cycling was similar to that of SAC305 BGA solder balls. Specifically, the BGA-like samples with Sn-Bi solder alloys exhibited an identical scenario of collapse behavior during thermal cycling.

The repeated reflow process can lead to a decrease in the height of the BGA solder ball and an increase in its width, resulting in a reduced distance between neighboring solder balls. This can increase the risk of short circuits or the susceptibility to dendritic growth (electrochemical migration), specifically in high electric fields and humid environments [28,29]. Additionally, in humid environments, flux residues can form an electrically conductive layer, increasing the leakage current [30], further contributing to the risk of short circuits.

## 5. Conclusions

This work provides a novel approach for measuring the BGA solder ball collapse behavior during repeated reflow with the aim of preventing manufacturing failures. The methodology enabled the measurement of the height change of BGA solder balls during reflow, as well as the identification of the formation of interconnections (short circuits) between the adjacent BGA solder balls. Two solder alloys, Sn63Pb37 and SAC305, were examined using this method, leading to several conclusions:

- The novel approach allows the investigation of the effect of repeated reflow on the collapse of BGA solder balls for various solder alloys and under different temperature profiles.
- The approach can identify an increased risk of short circuit occurrence at certain reflow temperatures.
- BGA solder balls of certain solder alloys, such as SAC305, are more likely to experience a greater degree of collapse after multiple reflows at lower temperatures than at higher temperatures.
- The collapse of solder balls can be controlled by a selection of reflow temperatures to avoid the formation of solder bridges. The suitability of either higher or lower temperatures is determined by the type of solder alloy.
- This approach is suitable for use in industry to measure BGA package collapse as a function of BGA package size, number of pins, and pin size, resulting in finding the appropriate temperature profile for soldering specific solder alloy type.

Although the TMA probe registers dimension changes in the whole BGA system, the expansion behavior of the BGA component and the substrate has been neglected in this work, as the expansion of the bare copper plate is negligible compared to the dimensional changes that occur during solder ball melting. However, in real systems, it would be necessary to consider the coefficient of thermal expansion for both the soldered BGA component and its substrate. This problem can be solved by optimizing the thermal profile, especially the time of preheat during soldering. The TMA equipment allows the creation of a program that can be modified for different variations. In this way, it will be possible to find the appropriate temperature profile for soldering specific solder alloy types, at which the difference in the thermal expansion of the substrate and the BGA package will be insignificant.

Another limitation is the maximum size of the evaluated sample, restricted by the capabilities of the TMA apparatus. Measurements cannot be performed on the entire manufactured board. However, as miniaturization progresses and the BGA package size decreases, this limitation may diminish over time.

## Data Availability

The raw data supporting the conclusions of this article will be made available by the authors on request.
